# Prescheduled Interleaving of Processing Reduces Interference in Motor-Cognitive Dual Tasks

**DOI:** 10.5334/joc.122

**Published:** 2020-09-30

**Authors:** Christine Langhanns, Hermann Müller

**Affiliations:** 1 Institute of Sport Science, Justus Liebig University Giessen, DE; 2 Nemolab, Justus Liebig University Giessen, DE; 3 Center of Mind, Brain and Behavior (CMBB), Universities of Marburg and Giessen, DE

**Keywords:** Cognitive-motor dual-tasking, treadmill walking, n-back task, resource theory, cognitive processing

## Abstract

Continuous motor tasks like walking have the potential to allow a dynamic allocation of processing resources when interrupted by intermittent cognitive tasks. The degree to which a successful interleaving of processing streams of both tasks is possible may depend on the temporal regularity of events. Fifteen subjects participated in an experiment where we systematically manipulated the regularity of stimulus onsets in a 2-back task relative to the step cycle. We tested three conditions where stimulus onset was always synchronous to a defined event in the stride (right heel strike, left heel strike, and midway between two heel strikes) and two conditions where the temporal location of the stimulus shifted from stride to stride. In order to test for potential effects of task difficulty, we also manipulated walking speed. We measured reaction times, accuracy of the reactions and several measures describing motor performance. There was no sign of task interference in these measures when stimuli always appeared at the same relative location within the step cycle. However, we observed prolonged reaction times when the stimulus came up earlier than expected. Surprisingly, in the other non-regular regime, where the stimulus appeared later than expected, reaction times were fastest. We interpret this result in the light of a prescheduled allocation of processing resources that is linked to the cyclic profile of processing requirements of the motor task.

## Introduction

Humans are limited in their ability to handle multiple tasks in parallel. Typically, at least one of the tasks shows performance decrements compared to a single-task control condition. Explanations commonly refer to a conflict at a central level of processing, where tasks compete for limited processing resources. This interference has been widely demonstrated in speeded reaction-time (RT) tasks where stimuli are presented in close temporal succession (Koch et al. 2018). Observed processing delays are used to infer how the temporal interleaving of processing streams is organized and how this organization depends on particular features of the tasks. Given the close temporal constraints of speeded RT tasks, subjects are very limited in implementing individual strategies in the temporal control of processing activities. However, dual-task interference in terms of performance decrements is also observed when tasks keep going for longer periods of time, like in many postural or locomotion tasks. Walking speed decreases when an additional cognitive task is performed concurrently, particularly in elderly persons but also in young and healthy adults (Yogev-Seligmann, Hausdorff & Giladi 2008). Given the strong empirical evidence for these phenomena, comparatively little is known about the underlying interference at the level of control processes (Wollesen & Voelcker-Rehage 2014).

Different from the aforementioned discrete RT tasks, locomotion tasks require a continuous processing and steady transfer of sensory input into precisely controlled motor commands. In addition, in a cyclic task like walking or running, one would expect that these processing requirements will also have a cyclic profile. The question then is, how is the processing of an intermittent cognitive secondary task interleaved with the cyclic regime of the continuous motor task? Given that little is known so far, we need to speculate about different possible alternatives. The following ideas how we might conceive processing in continuous cyclic motor-cognitive dual tasks is based on the concept of shared resources (Kahneman 1973). Being aware of the perpetual discussion about the pertinence of the concept of a *resource* in explaining dual-task effects, we will try to confine the assumptions implied with the use of that term to only a few aspects: i.) We think of a resource as a capacity allowing successful dealing with processing requirements of behavioral tasks, ii.) the allocation of this capacity to a given task is scalable and its amount can be described as a quantity, iii.) performance in a task depends on the allocated quantity, and iv.) the overall quantity is limited. Based on these assumptions, we will use the concept in a purely functional model. We are aware that, in this vein, we exclude interesting physiological and mechanistic questions, but these are not decisive for the expectations derived from our simplified models.

**Figure 1:**
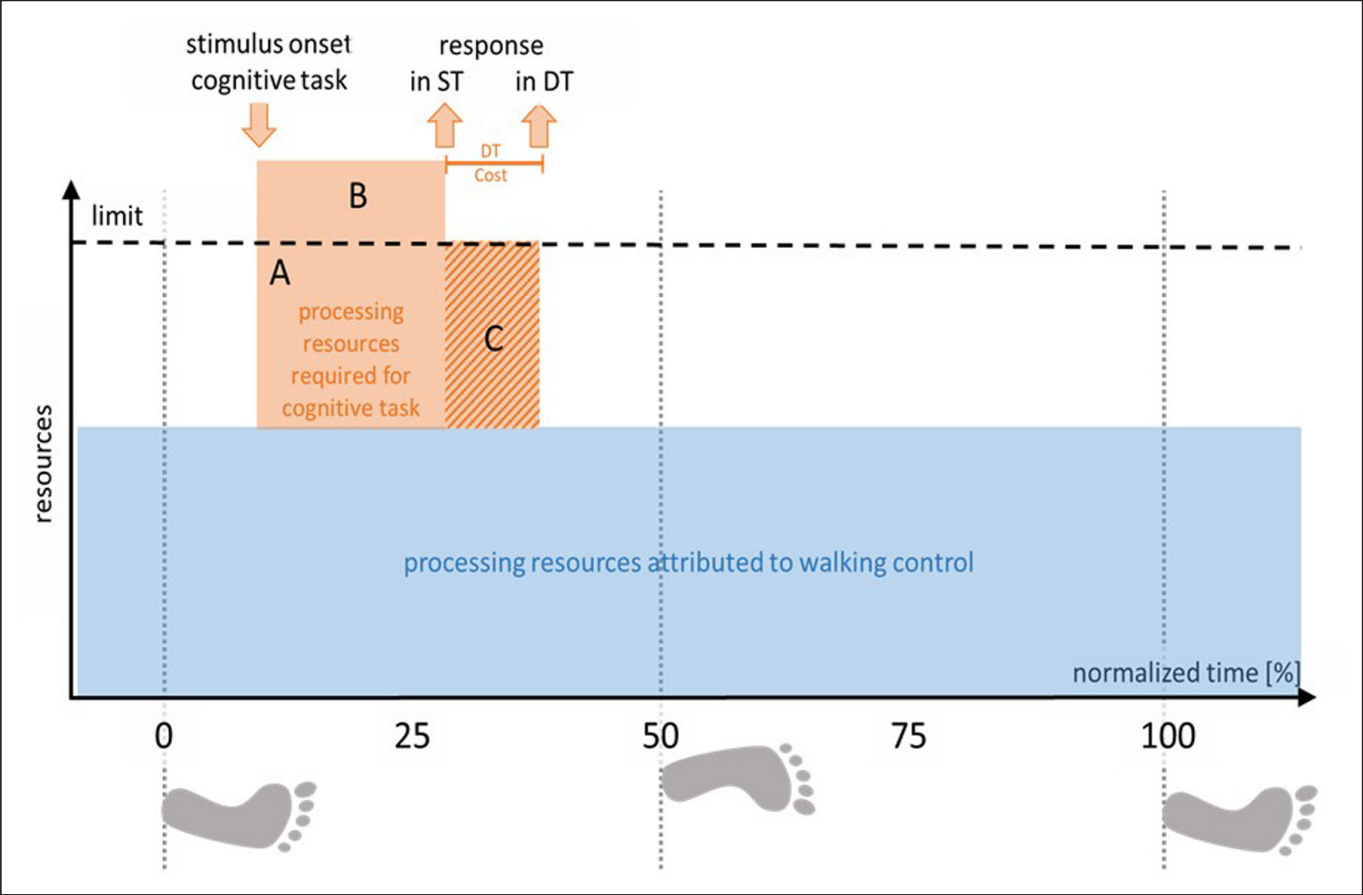
**Performance decrements in the cognitive task due to resource limitations.** A continuous motor task (in our example: walking) continuously draws on limited resources throughout the entire movement cycle (blue rectangle). If an intermittent cognitive task is triggered by a stimulus at a given moment in that cycle, both tasks have to share the limited amount of processing resources (vertical axis). For simplicity, we restrict this illustration here to the case that motor processes claim a constant amount of resources and that only the cognitive task is affected by this limitation. Yet, we will loosen these restraints later. Optimal processing of the cognitive task would require a given processing capacity (amount × duration), here represented by the area A + B. However, due to the overall limitation and the parallel demand of the motor task, only the amount A is currently available. If now, the task needs to be as fast as under typical single-task (ST) conditions, quality of task achievement will drop due to the loss of B. Alternatively, the duration of task processing might be prolonged, allowing temporally extended use of resources (duration of C) to compensate for the shortfall of B. Typically, this temporal delay is taken as measure for the amount of task interference in the latter case.

Here, we assume that both tasks draw at least to some degree on the same limited processing resources at a certain, yet unspecified level of control. As stated in iii.), performance in each task depends on the amount of resources available for that task. Accordingly, if information processing in a given task has to be performed with insufficient capacities, the completion of that task will either be delayed or its quality be diminished compared to a situation where sufficient resources are available, e.g. in a single-task situation (for an illustration see Figure 1).

### Fixed temporal binding to motor task

**Figure 2:**
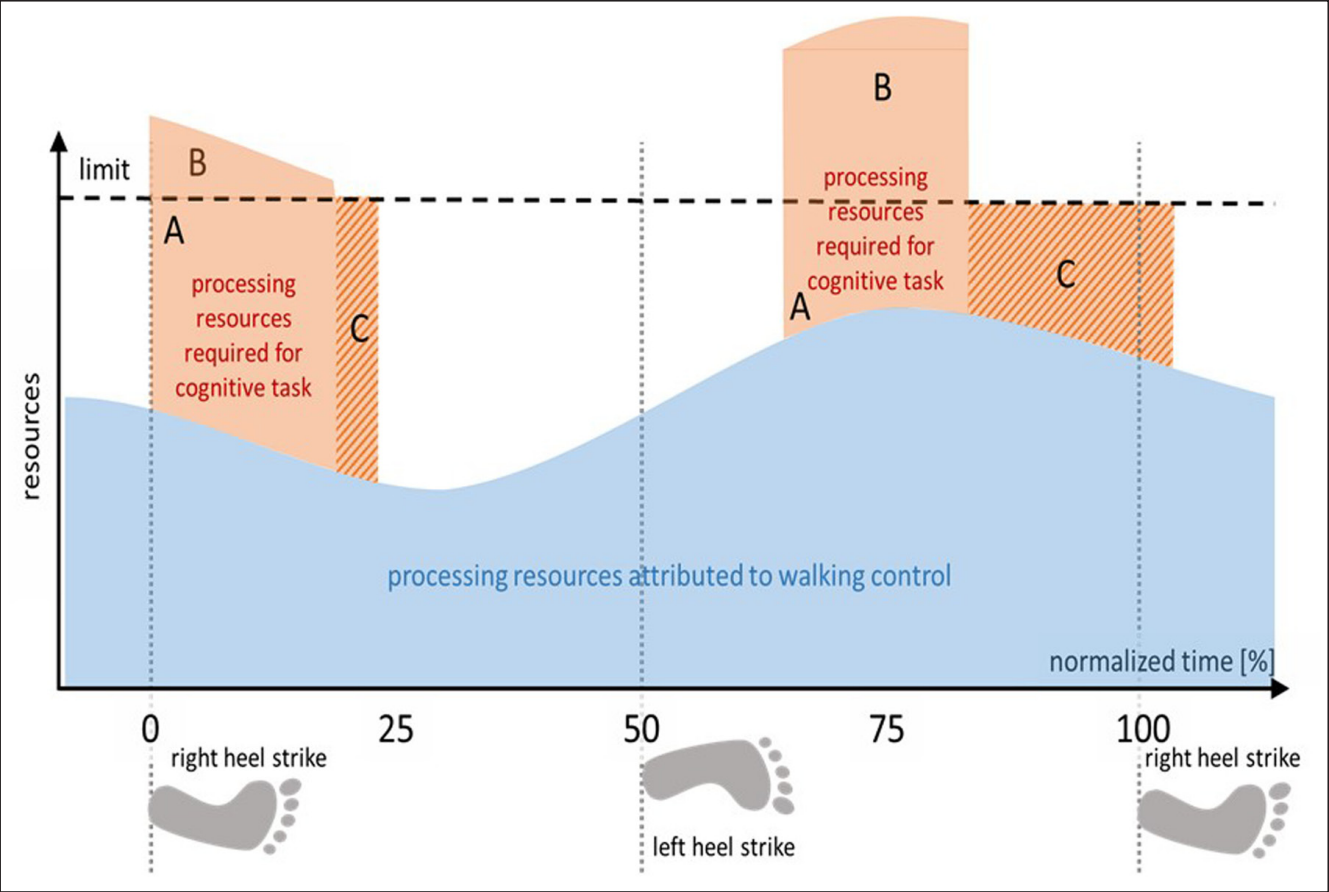
**Fixed cycle-linked alteration of processing requirements of the motor task.** The motor tasks requires different amounts of processing resources at different moments in the cycle. The cognitive task is presented at different temporal locations in that cycle (stimulus onset at 0%, resp. 60%). Dependent on the amount of unmet requirements (B), it takes longer (larger horizontal extension of compensatory process supply C in the rightward [60%] location) to complete the cognitive task. Differences in response delays are thus informative about changes in processing requirement of the motor task across the motor cycle.

We now introduce the additional assumption that processing requirements for the motor task vary across the cycle and that this regime is strictly temporally linked to the ongoing cyclic activity. Presenting the cognitive task always at a fixed temporal position in that cycle allows the determination of the size of interference (SoI) in terms of quality loss and/or delay for that particular location. We can measure SoI for different temporal positions (t_1_, t_2_, t_3_) and compare values. We might observe that tasks interfere while SoI is different depending on the temporal position of the cognitive task, e.g. SoI(t_1_) > SoI(t_2_). In that latter case, we would reason that the processing of the motor task is more demanding at moment t_1_ compared to t_2_, or at least, that processing of both tasks cannot be interleaved as easily at t_1_ as at t_2_ (see Figure 2).

Yet, it is also possible that we do not find any differences in SoI across different positions in the motor cycle, even though all SoIs are larger than zero. In this case, we would be inclined to deduce that the motor task requires constant amounts of processing resources throughout the whole cycle. However, the same result could also be expected, if we dismiss the prior assumption of a fixed temporal link between motor activity and processing requirements and allow temporal flexibility. Despite the observed prioritization of postural and locomotor tasks (Lövdén et al. 2008), the degree to which this priority actually dictates resource allocation might change over time, probably partly modulated by the requirements of the cognitive task. Locomotor tasks are highly practiced, involving control at different hierarchical levels. At least some of those are considered as being *automatic*. This means that they operate without immediate involvement of higher-order control entities, i.e. those entities that are shared with the secondary task. Given that automatic control secures the basic flow of events, higher-order control may only be required intermittently, with the actual temporal locus of this involvement being more or less flexible. If this were the case, subjects could temporally shift processing resources according to the needs of the cognitive secondary task. There are different ways of how this shifting could be organized.

### Triggered Interruptions

**Figure 3:**
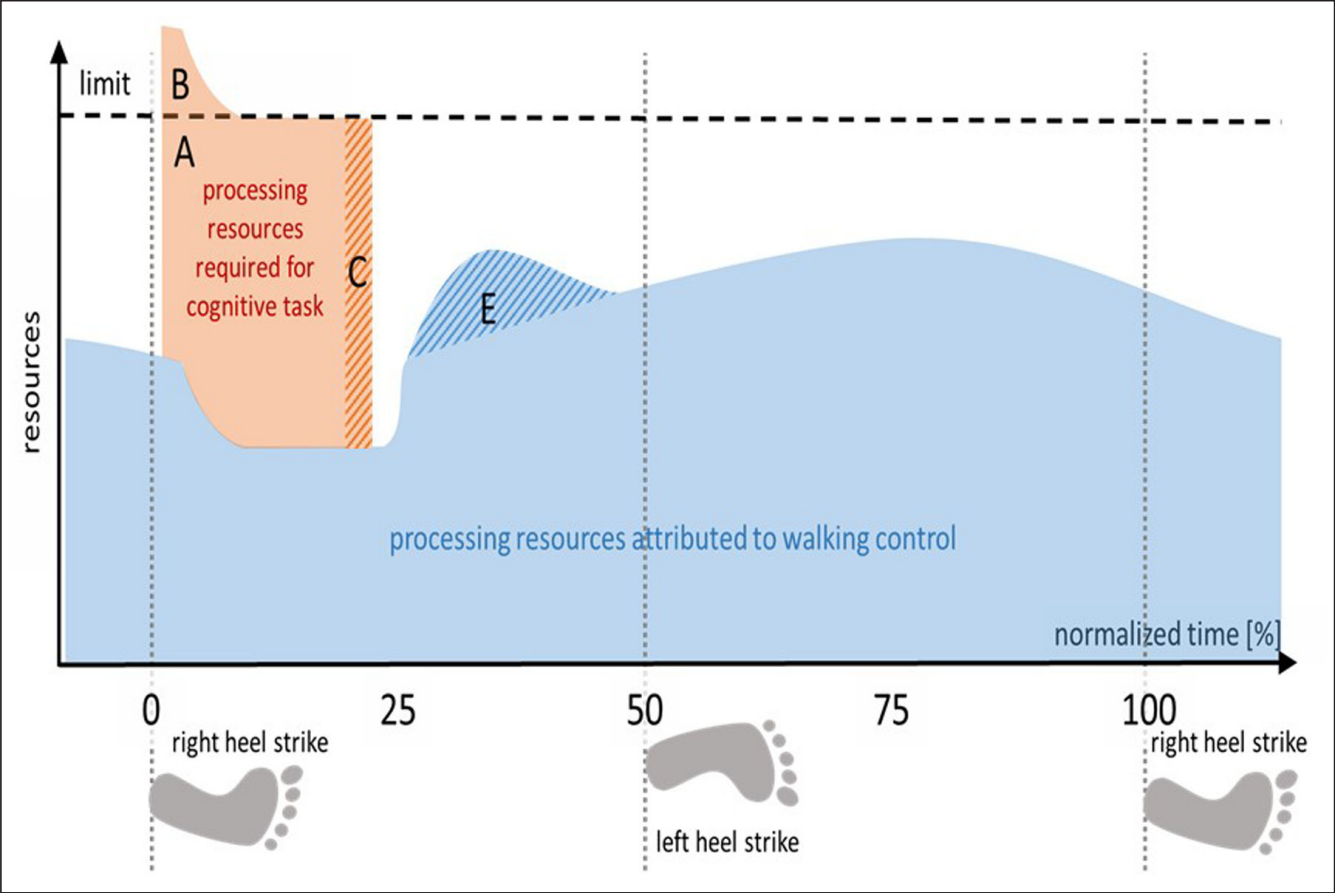
**Triggered interruption of motor task processing.** An unexpectedly occurring cognitive secondary task interrupts processing of the motor task. The shift of resource allocation cannot be effected immediately. Dependent on the amount of unmet requirements (B), accumulated during that shifting period, it takes longer to complete the cognitive task. Response delays (horizontal extension of C) inform about the quickness of the shifting in resource allocation. Whether motor performance is also affected depends on whether the interrupted motor processes can easily be shifted to a different location (E).

For the purpose of illustration, we pick a moment, where higher-order entities are currently involved in the control of the motor task. If now, a stimulus triggers a secondary task requesting additional processing capacity, the system has two options. First, the motor process is allowed to keep on occupying the resources until the end of the current intermittent control episode. This is equivalent to the *Fixed Temporal Binding* case we have discussed previously. Accordingly, we would expect to see performance decrements primarily in the cognitive task, probably in terms of a delayed response. Alternatively, the control system could interrupt the motor control processes as soon as possible to free resources for the cognitive task. However, this shifting of resource allocation might not be feasible instantaneously, but will require some time. Dependent on the rapidness of the shifting process, decrements in cognitive performance are also possible. In addition, negative effects may also appear in the motor task. We expect larger decrements, if motor task processing is interrupted at a critical moment, in particular, if interrupted processes cannot be shifted to a different location without performance loss (see Figure 3).

### Scheduled Interruptions

**Figure 4:**
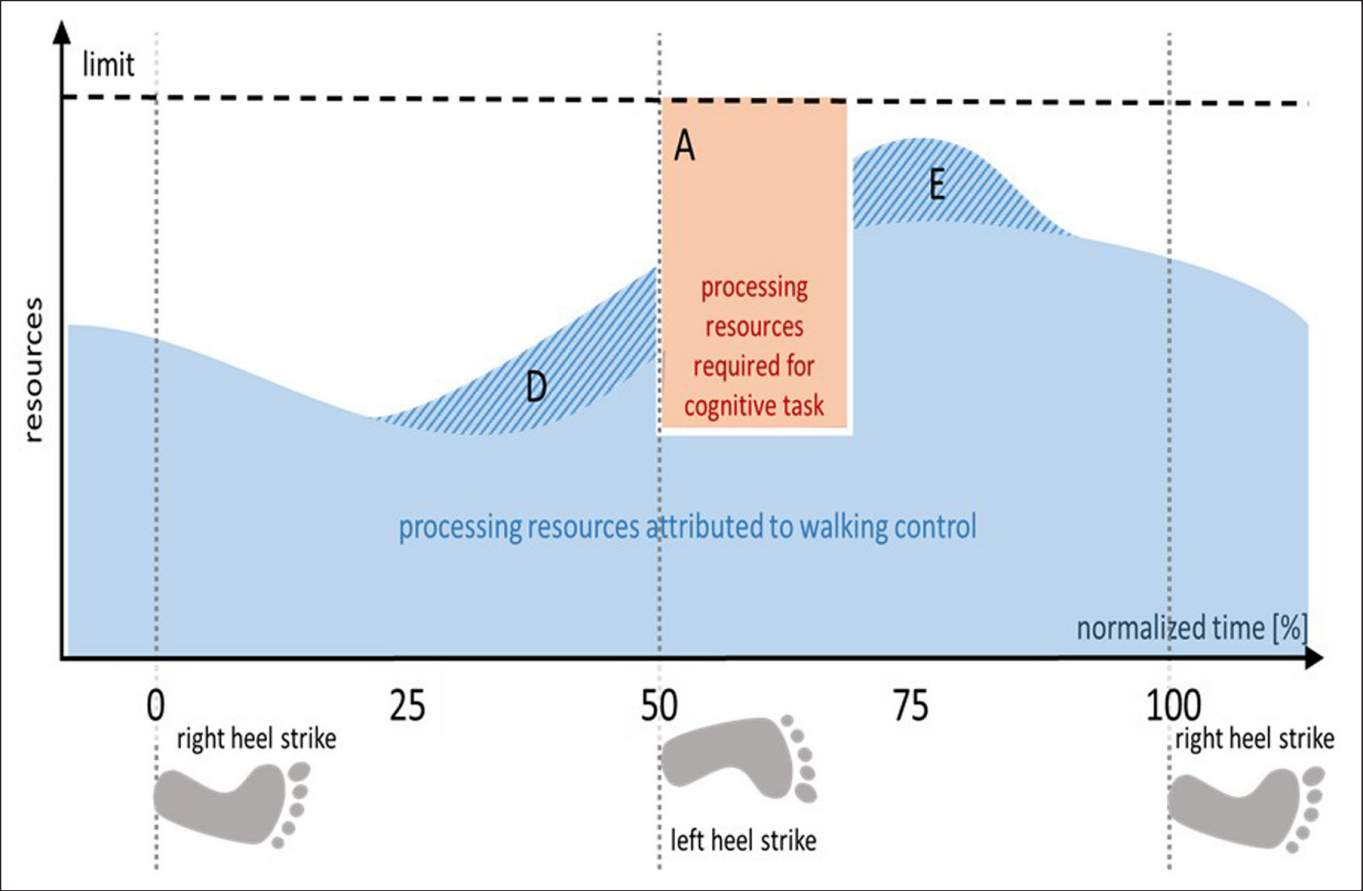
**Scheduled temporal interleaving of processes.** The secondary task has to be processed at a predictable temporal position in the motor cycle. In order to free sufficient capacity (A) to execute the cognitive task with performance loss, part of the motor processing is shifted in time. This could either be before (D) or after (E) the period of cognitive task processing.

In the examples discussed so far, we have presumed that the subject does not know the temporal location of the cognitive task *a priori* and is more or less ‘surprised’ by it. This would be different, if the secondary task is temporally linked to a defined temporal position in the motor cycle. Being familiar with the time course of the upcoming processing requirements, subjects could anticipatorily shift the intermittent central control of the motor task away from the critical moment, when the cognitive task is expected to come up. This is linked to the idea of *time-based expectancies* (Volberg & Thomaschke 2017). If resource allocation could be prescheduled, neither an ongoing motor process would have to be interrupted prematurely, nor would the cognitive process have to wait (see Figure 4). If such a preplanned temporal interleaving of processing streams would be possible, interference should be reduced or even absent in situations where both tasks are linked in a fixed temporal regime. Particularly, removing this regularity should increase SoI.

Based on a resource allocation logic, we have discussed three different situations. Each of these will reveal itself by a characteristic profile of interference in a (rhythmic) motor- (intermittent) cognitive dual task.

In case of a *Fixed Temporal Binding* of motor processing to the motor cycle, we expect that:

**Table:** 

A1-	interference is only visible in the cognitive task,
A2-	interference scales with the overall difficulty (e.g. speed) of the motor task,
A3-	the magnitude of interference might be different for different temporal locations.

When task processing is similar to a *Triggered Interruption*, we expect that:

**Table:** 

B1-	interference may be observed in both tasks, cognitive and motor,
B2-	interference scales with the overall difficulty (e.g. speed) of the motor task,
B3-	the magnitude of interference might be different for different temporal locations.

If task processing is modelled as *Scheduled Interruption*, we predict that:

**Table:** 

C1-	interference might be reduced or even completely absent in case of an obvious temporal regularity, however…
C2-	becomes larger, when the regularity is removed.
C3-	No clear predictions can be made on the dependency on changes in difficulty of the motor task within (different moments in the cycle) and across motor tasks (e.g. different speeds).

Despite the fact that motor-cognitive dual tasks are widely studied, only a few have actually looked into the temporal interleaving of processing for both subtasks. Abbud, Li, and DeMont (2009) observed different amounts of interference (changes in EMG-amplitudes in walking muscles) dependent on the temporal location of cognitive task onset (B3) while the cognitive task showed no interference effect. Other studies, however, also reported effects on the cognitive side. According to A3, reaction times were longer when the cognitive task was executed during single compared to double stance phase (Lajoie et al. 1993). In another study, reaction times were longer for slow compared to faster overground walking (Lajoie et al. 2015). Following A2, this would be interpreted as indication that slow walking is not necessarily the less resource-demanding mode.

In the following experiment we will build on this existing work and investigate dual-task interference while subjects walk on a treadmill and execute an intermittent cognitive task. Both tasks are performed as single-tasks but also in a dual-task condition to quantify the amount of dual-task interference. The cognitive task in this study requires reactions to stimuli in a *2*-back task which are either systematically linked to a defined temporal location within the cyclic motor activity or are shifted to a different location from stride to stride. In addition, we manipulate task difficulty by testing at different walking speeds, which affects both tasks. Given that we present *2*-back stimuli linked to a fixed event in the walking cycle, higher walking speeds do not just increase cadence, frequency of *2*-back items will also rise. Yet, we cannot predict the frequency effect on task processing *a priori*. Even though, a large number of studies investigated working memory by manipulating working-memory load and stimulus modality under single and dual-task conditions, there is still little systematic research on how *n*-back task properties like stimulus frequency affects performance (Coulacoglou & Saklofske 2017).

## Method

### Participants

We calculated the required sample size to achieve a power of 0.8 given α = 0.05 and an effect size of 0.5 by using G*Power software (version 3.9.1.2; Faul, Erdfelder, Lang, & Buchner 2007). Accordingly, 15 healthy students (8 female, 7 male; mean age: 23.1 ± 3.4 years) participated in this study. The cohort exhibited an average score of 92 ± 13.2 according to Oldfield’s handedness inventory (1971) indicating right handedness. All participants declared physical, neurological and psychiatric healthiness and normal or corrected to normal vision. Participation was compensated by either a fixed course credit or monetary reward. The local Ethics committee approved the experiment and all participants gave written consent.

### Motor task

In the *Walking* condition, subjects walked (freehand) on a treadmill (h/p/cosmos Sports & Medical GmbH, Nussdorf-Traunstein, Germany), wearing a safety belt. They walked at speeds of 3, 4, or 5 km h^–1^ for 60 s while looking at a computer screen at a defined distance (approx. 1.5 m). The walking speeds were selected in order to comply with two basic requirements: firstly, the speed range should introduce sufficient variation in task difficulty, and while secondly, walking speeds should remain in the range of preferred treadmill walking speeds of young and healthy adults (Regnaux, Robertson, Smail, Daniel, & Bussel 2006, Kurosawa 1994).

### Cognitive task

We used a *2*-back task with visual stimuli. Letters were presented for 450 ms in the center of the screen. The task required a choice reaction by pressing one of two predefined buttons on a reaction-time box (Laboratory of Brain Processes, Columbus, USA) with either the index finger or the ring finger of the right hand. One finger indicated a *2*-back matching, whereas the other indicated a mismatch. Finger-button coding was counterbalanced across subjects. In only around 15% of the trials, a *2*-back matching stimulus was presented. Thus, one finger had to be used more frequently. Potentially, as a result of this manipulation, more cognitive effort is required to inhibit the less frequent reaction for a mismatch. We therefore tested for differences between matches and non-matches. To avoid muscular fatigue in arm and hand by simply holding the reaction-time box, we fixed the box to the holding hand with an elastic tape. This allowed subjects to loosen the grip without losing the box. In the cognitive single-task condition, subjects were instructed to respond as fast and accurate as possible while sitting on a chair without arm rests.

### Motor-cognitive dual-task

Stimulus onset of the *2*-back task relative to the step cycle was systematically manipulated. Three fixed temporal locations in normalized time relative to the right heel strike were used in the experiment. In the *0%* condition, stimulus onset was simultaneous to the right heel-strike. In the *50%* condition, stimulus onset was midway between two right heel-strikes, which was approximately the moment of the left heel strike. In the *75%* condition, stimulus onset was midway between a left heel-strike and a right heel-strike.

Irrespective of the actual location, stimuli were only presented every second stride. Since stimulus frequency was linked to step frequency, inter-stimuli intervals were different across walking speeds. The range of resulting stimulus frequencies is listed in Table 1. As an adequate single-task control condition for each of the three velocities, subjects performed the *2*-back task while sitting also with three different frequencies adapted to cadence. Each subject’s preferred cadence for a given velocity was individually determined in single-task walking pretests on each test day.

**Table 1:** Stimulus frequencies for different treadmill speeds.

Speed [km h^–1^]	Range [Hz]	Mean [Hz]	Standard deviation [Hz]

3	0.339–0.446	0.397	0.025
4	0.388–0.482	0.445	0.023
5	0.436–0.507	0.478	0.020

In addition to the three fixed temporal positions, we introduced two further conditions, where the *2*-back stimulus permanently changed its temporal position, however not completely irregular.

In the *Later* condition, the first stimulus in each trial always appeared at 5% of the normalized stride time. Each following stimulus was delayed by additional 5% stride duration, leading to a sequence of stimulus onsets of 5, 10, 15, …, 100%. If a trial included more than 20 stimuli, this sequence commenced from the beginning. Equivalently, in the *Earlier* condition, the first stimulus onset was at 45% of the normalized stride time and was shifted ahead by 5% in each following repetition.

### Procedure

Testing started with a familiarization session one or two days before the actual experiment. Within this session, subjects practiced walking on the treadmill at each of the tested speeds. They also practiced the *2*-back task until they reached a performance level of at least 80% correct responses in three consecutive trials.

**Figure 5:**
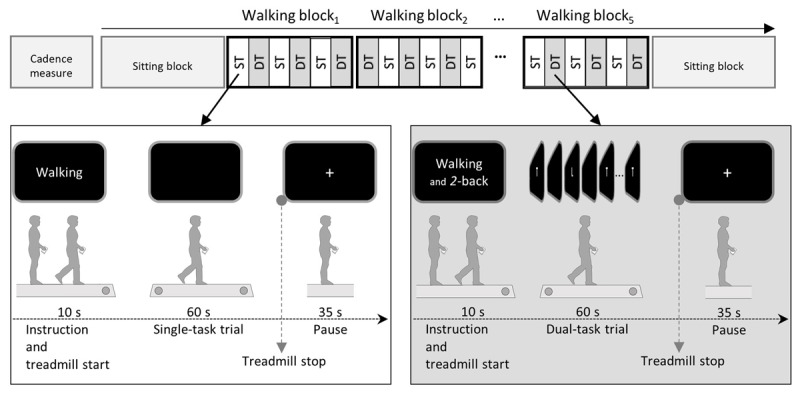
**Experimental procedure.** Top panel shows the sequence of blocks within a testing day. Each day started with an ST-*Walking* test to measure subject’s preferred cadence for the given speed. The following sequence of seven test blocks starts and ends with a *Sitting* block. In between subjects perform five *Walking* blocks for the different SORs. Sequence of SORs is counterbalanced across subjects. Each block consists of three ST and three DT trials in alternation. Boxes below depict the timeline of an ST trial (left) and a DT trial (right).

In the actual experiment, each subject was tested on three consecutive days, with walking speed fixed within day, yet varying across days. As depicted on Figure 5, each day started with simple treadmill walking to determine cadence and based on this, the individual stimulus frequency for the *2*-back task in the ensuing *Sitting* condition. Please note, this was conducted for the cognitive single-task condition only. Under dual-task condition, SOR was determined online by processing the individual walking pattern. The last block on each day was also performed as *Sitting* condition. In between, subjects performed five *Walking* condition blocks. Each block contained three dual-task trials (walking & *2*-back) alternated with three single-task walking trials. Each trial lasted 60 s with 35 s inter-trial pause for cognitive and/or physical recovery. Each 60 s trial was preceded by a 10 s period in which test instructions were presented and the treadmill was started and ran up to the predefined speed. Overall daily testing time was about 80 min. The order of speed and of walking conditions were separately randomized across subjects in order to counteract systematic sequence effect.

### Motion capturing

Treadmill walk was monitored using a VICON (Oxford, UK) motion capturing system and the corresponding data acquisition software Nexus 1.8.5. Six passive reflective markers were attached according to the Plug-in Gait model to heel, forefoot, and calf of participant’s left and right leg. The spatial positions of the markers were recorded with 5 cameras (2 cameras of type MX and 3 cameras of type MX-3) with a sampling rate of 100 Hz, preprocessed and stored with VICON DataStream SDK 1.3.0 software. Matlab 8.1 (MathWorks Inc., Nattick, USA) programs were used for online access to this data stream, in order to check gait cycles, determine stimulus onset, and present stimuli (PsychToolBox 3.0).

Kinematic raw data from motion capturing was filtered using a 2^nd^ order Butterworth low pass filter (cut off frequency = 5 Hz). Gaps were refilled using a spline cubic interpolation. Right and left heel markers were used to detect heel strikes, in order to calculate the averaged stride duration (SD) and stride length (SL). We also calculated Coefficients of Variation (CV = mean/standard deviation) for both parameters (SD_CV_, SL_CV_). The subtraction of dual-task performance from single-task performance represents the amount of dual-task costs for each walking parameter (ΔSD, ΔSL, ΔSD_CV_, ΔSL_CV_).

### Preprocessing of RT data

**Figure 6:**
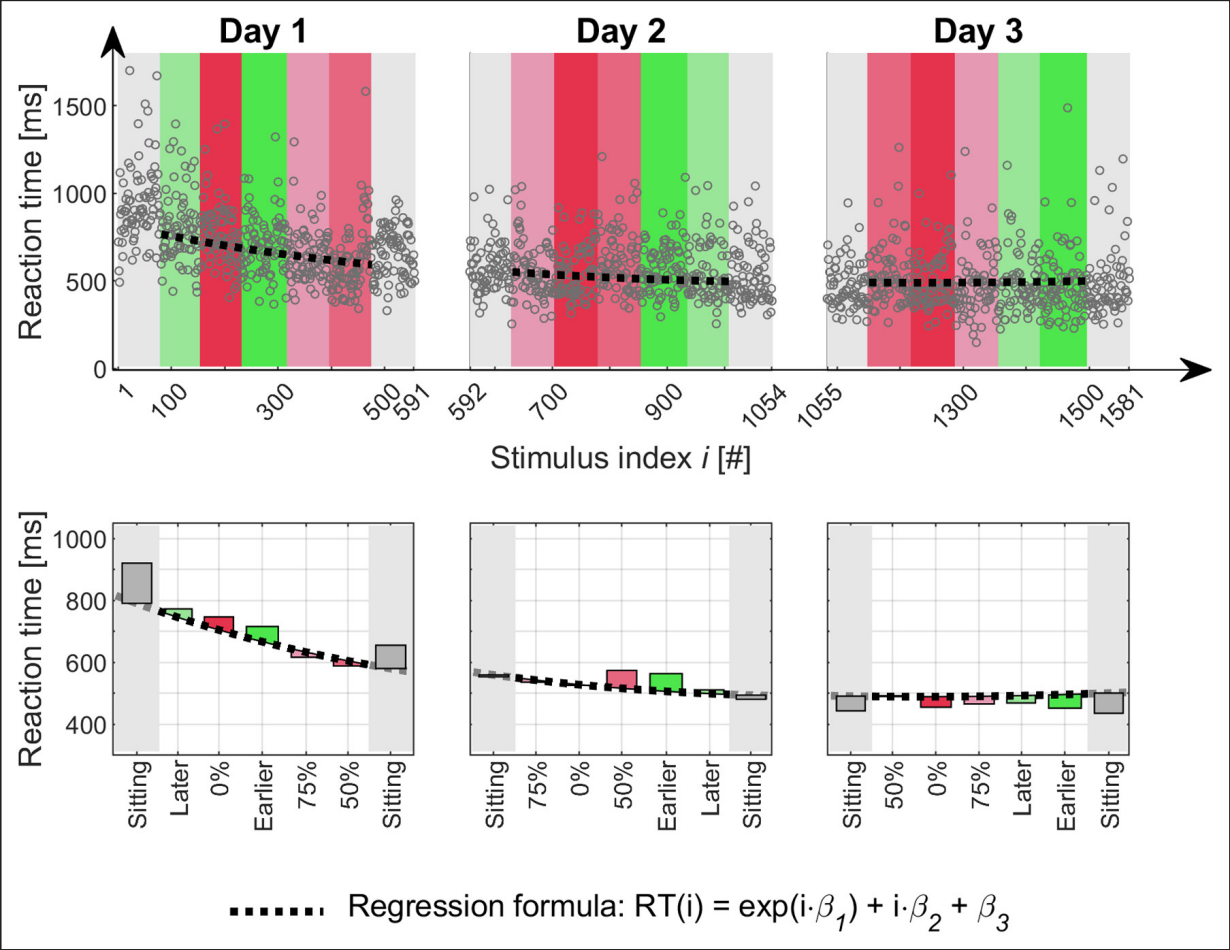
**Exemplary data of a single subject.** The upper row shows reaction times recorded over time and the calculated regression function (black dotted line). Background colors indicate the *Stimulus Onset Regimes* decoded in the according boxes in the lower row. Bars in the lower row represent distance of median to predicted mean performance.

Performance typically improves with increasing experience, hence, in our *2*-back task, continued practice should decrease RT and/or increase accuracy, bringing in additional error variance into these dependent measures. In perfectly counterbalanced designs, this error variance does not systematically distort the results, however, it still reduces statistical power. Therefore, we tried to eliminate learning related variance by a non-linear regression (Newell, Liu, & Mayer-Kress 2001) and determined the regression coefficients individually for each subject (see Figure 6). We further analyzed the residuals of the RT data, relative to this regression. In order to have an estimate of the average performance that is robust against the typical skewedness of RT data, we relied on the Median. For each subject and each condition, median RT_residual_ per trial was calculated and averaged across trials.

*Task Accuracy* (ACC) was calculated as the ratio of number of correct responses and number of required correct responses within one trial. These ratios were then averaged across trials. Different from RTs, we did not observe a systematic improvement in accuracy over time. In order to check for possible effects of response frequency, we did not only look at the integrated accuracy across all trials (ACC_total_), but also analyzed responses to matching (ACC_match_) and non-matchings (ACC_non-match_) separately.

### Statistical analyses

The influence of the independent variables *Walking Speed* (3/4/5 km h^–1^) and *Stimulus Onset Regime* (*SOR*: 0%/50%/75%/Earlier/Later) on the dependent variables RT_residual_ and ACC for the cognitive task were tested by separate repeated measures ANOVAs. Similarly, motor performance parameters (SD, SL, SD_CV_, SL_CV_) and dual-task costs in motor performance (ΔSD, ΔSL, ΔSD_CV_, ΔSL_CV_) were tested by repeated measures ANOVAs for effects of *Walking Speed* and *SOR*. All inferential statistics were executed with JASP (version: 0.11.1, University of Amsterdam, The Netherlands).

## Results

### Cognitive performance

Figure 6 depicts cognitive task performance (RT) of an exemplary subject. RTs show a typical positive skew and decrease in value with stimulus number according to an exponential function. Differences between regression line and the median within each block is taken as measure for the relative performance effect (RT_residual_) of the particular condition.

**Figure 7:**
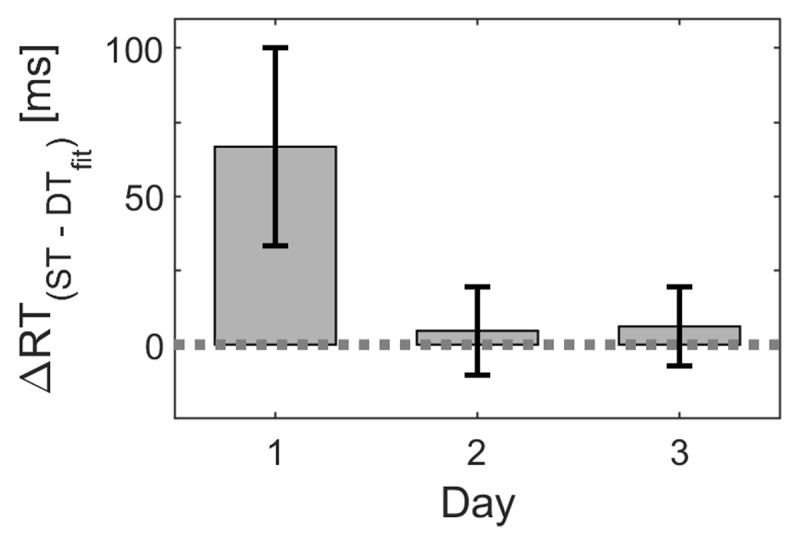
**Relative reaction times in the *Sitting* condition.** Means and standard errors including 95% confidence interval are presented separately for the three testing days.

On the first day, particularly in the first block, RTs while sitting were relatively long, compared to the walking condition. Later, after being sufficiently acquainted to the task, RTs while sitting and walking are comparable (see Figure 7). In days 2 and 3, there is no general interference effect when executing the cognitive task while walking on the treadmill. However, there are differences between walking conditions.

**Figure 8:**
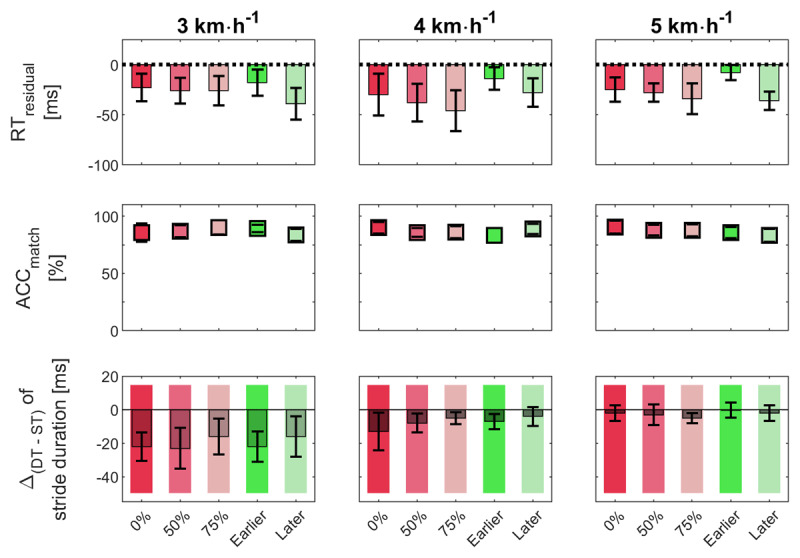
**Result overview of cognitive and motor performance for different *Stimulus Onset Regimes* and *Walking Speeds*.** Cognitive performance is depicted in the upper two rows. The first row represents the residual reaction times (RT_residual_). The second row shows the accuracy measure for *2*-back matchings (ACC_match_). The third row represents the difference between dual-task and single-task [Δ_(DT-ST)_] performance in step duration. Negative values indicate shorter durations under dual-task condition. Mean values of dependent variables across conditions. Error lines represent standard error including 95% confidence interval.

**Figure 9:**
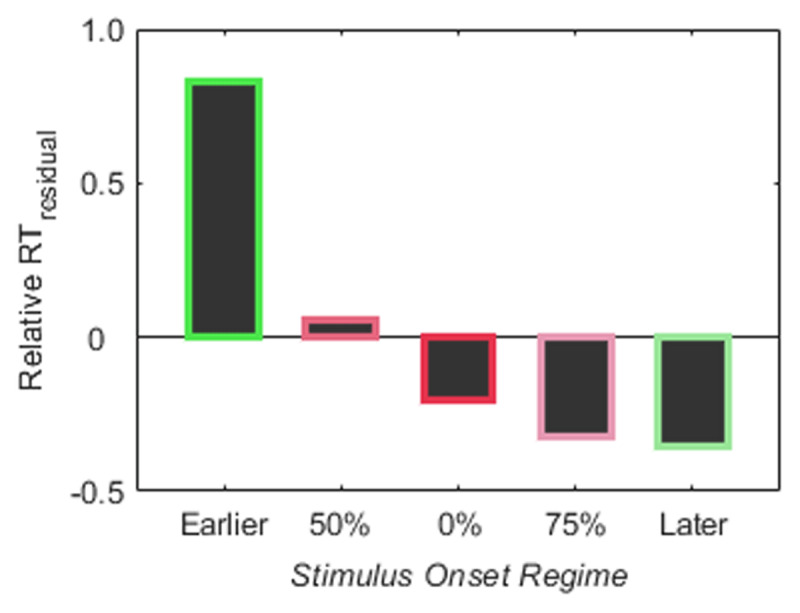
**Profile of relative RTs for different *SOR* conditions.** RT_residual_ values are integrated across walking speeds and z-transformed.

A 2-way (factors: *Walking Speed* [3,4,5 km h^–1^], *SOR* [0%, 50%, 75%, Earlier, Later]) repeated measurements ANOVA for the dependent variable RT_residual_ showed a significant main effect of factor *SOR, F*(2.864,40.103) = 5.621, *p* = 0.003, *η*^2^ = 0.076. Means and dispersion of RT_residual_ values are presented in Figure 8 (upper row). *Post hoc* comparisons revealed longer RTs, when the stimulus appeared systematically earlier in the walking cycle, compared to the systematically later stimulus onset, *t*(14) = 4.536, *p* = 0.005, Cohen’s *d* = 1.171 and the *75%* condition, *t*(14) = 4.102, *p* = 0.010, Cohen’s *d* = 1.059. No further effects were significant. There is neither a main effect for factor *Walking Speed, F*(1.403,19.635) = 0.519, *p* = 0.540, *η*^2^ = 0.006, nor an interaction, *F*(4.518,63.249) = 1.030, *p* = 0.404, *η*^2^ = 0.028. We also do not find any significant (all *p*’s > 0.05) differences in RT_residual_ comparing reactions to matching stimuli with reactions to non-matching stimuli. In the absence of a *Walking Speed* effect, we accumulated values of RT_residual_ across walking speeds in order to depict RT characteristics across speeds (see Figure 9). Since these RT values come from conditions with different underlying variability, we z-transformed data before integrating them. The resulting profile depicts the dependency of RTs on *SOR* conditions. RTs are longest in the Earlier and shortest in the Later condition, with all other conditions in between.

We entered all ACC measures into equivalent ANOVAs. Figure 8 (mid row) depicts the results for the reactions to matching stimuli (ACC_match_). These represent the case of the least frequent response, which might be expected to be the most sensitive parameter. However, neither main effects nor interaction were significant. *SOR, F*(2.44,34.18) = 1.015, *p* = 0.386, *η*^2^ = 0.014; *Walking Speed, F*(2,28) = 0.011, *p* = 0.989, *η*^2^ < 0.001; *SOR* × *Walking Speed, F*(4.34,60.75) = 1.628, *p* = 0.174, *η*^2^ = 0.038. Test statistics of the other ACC measures confirm this result, all *p*-values > 0.168.

### Motor performance with additional cognitive load

We determined four stride parameters: Means of Stride Duration (SD), and Stride Length (SL) and the corresponding coefficients of variation (SD_CV_, SL_CV_) within each trial. Note that these parameters are not completely independent. As expected, SD and SL showed a reciprocal dependency on *Walking Speed*, whereas variability measures had parallel trends. SL_CV_ decreased with increasing walking speed. SD_CV_ was also largest at the slowest speed, while the two faster speeds were not significantly different. The different onset regimes (factor *SOR*) did not show any effect on the walking parameters. Dual-task costs, i.e. changes of walking parameters from single to dual task, can be observed for the factor *Walking Speed* in ΔSD (see Figure 8 lower row) and ΔSL. Steps were much shorter under dual-task condition for the slow speed compared to both faster speeds. Variability measures (ΔSD_CV_, ΔSL_CV_) remained less affected by *Walking Speed* and/or *SOR*. The results of inference statistical tests (2-way ANOVAs; factors: *Walking Speed* [3, 4, 5 km h^–1^], *SOR* [0%, 50%, 75%, Earlier, Later]) are listed in Table 2.

**Table 2:** **Results of ANOVA-tests for walking parameters.** SD = stride duration, SL = stride length, SD_CV_ = coefficient of variation of SD, SL_CV_ = coefficient of variation of SL, Δ = single minus dual-task performance in the dependent parameter, SOR = Stimulus Onset Regime.

Dual-task…	Parameter	Factor	*F*	*η*^2^	*p*

…walking pattern	SD	Walking Speed	332.797	0.679	<0.001
		SOR	0.575	<0.01	0.618
		Walking Speed × SOR	1.404	<0.01	0.240
	SD_CV_	Walking Speed	21.766	0.267	<0.001
		SOR	0.477	<0.01	0.711
		Walking Speed × SOR	0.518	<0.01	0.721
	SL	Walking Speed	325.299	0.537	<0.001
		SOR	1.352	<0.01	0.274
		Walking Speed × SOR	1.877	<0.01	0.122
	SL_CV_	Walking Speed	38.420	0.404	<0.001
		SOR	2.672	0.015	0.099
		Walking Speed × SOR	1.683	0.018	0.207
…costs	ΔSD	Walking Speed	18.121	0.194	<0.001
		SOR	0.928	0.011	0.423
		Walking Speed × SOR	0.831	0.015	0.458
	ΔSD_CV_	Walking Speed	5.203	0.117	0.036
		SOR	0.062	<0.01	0.978
		Walking Speed × SOR	0.835	0.013	0.514
	ΔSL	Walking Speed	8.184	0.105	0.002
		SOR	1.131	0.009	0.351
		Walking Speed × SOR	0.340	0.006	0.864
	ΔSL_CV_	Walking Speed	0.591	0.010	0.561
		SOR	0.729	0.014	0.467
		Walking Speed × SOR	0.376	0.011	0.748

## Discussion

In this study we aimed to investigate how different regimes of temporal coupling between a continuous motor task and an intermittent cognitive task affect the amount of task interference. So far, researchers avoided rhythmic linking of both tasks in such motor-cognitive dual tasks by presenting stimuli for the cognitive task at random, or at least unpredictable moments. These studies show mixed results. In some cases, interference is only visible in the motor task, which is often interpreted as motor task prioritization. In other cases, performances show signs of interference in both tasks. However, there are also studies showing no interference at all. Given this unclear state of knowledge, we used a resource-logic to derive predictions on how the amount of interference might be critically modulated by the existence of a predictable temporal relationship between both tasks. In order to test these predictions, we systematically manipulated the temporal regularity between stimulus onset in a *2*-back task and the step cycle while walking. To control for effects of overall task difficulty, we also manipulated walking speed and, linked to it, stimulus frequency.

Our major finding is a decreased cognitive performance, i.e. increased RT, in the condition when subjects encounter a stimulus earlier than expected. This performance decrement cannot be explained as a trade-off between speed and accuracy. Response accuracy was not increased in exchange.

However, before discussing this effect in the context of our explanatory models, we will discuss the impact of our difficulty manipulation. It is assumed that higher motor difficulty based on movement speed could leave less resources for the cognitive task and thus reduce cognitive performance (Wollesen & Voelcker-Rehage 2014). However, we observed walking-speed effects only on several walking parameters but not on cognitive performance measures. Wrightson, Ross, and Smeeton (2016) used a visual *2*-back task with constant stimulus frequency for preferred and very slow walking. They also did not find an effect of walking speed on the accuracy of the cognitive task. However, we cannot say whether RT may be affected, because these were not recorded in that study. Kline, Poggensee, and Ferris (2014) also did not find an influence of walking speed on performance in the Brook’s task while using a constant stimulus frequency. In line with these reports, Beurskens and Bock (2012) argued that working memory tasks like *n*-back tasks would not interfere with walking at all. In contrast, other researchers reported that slow walking would need more attention, which negatively affects serial subtraction performance (Nascimbeni, Minchillo, Salatino, Morabito, & Ricci 2015), and in the same line of reasoning, faster walking could boost serial subtraction (Penati, Shieppati, & Nadone 2020). The presence or absence of a speed effect in the cognitive task might be explained by different characteristics of the cognitive task, with the *n*-back task being more representative of a discrete, strictly time-limited task while serial subtraction can be considered as a more continuous task requiring processing over an extended period of time, which might allow larger temporal flexibility.

In line with these findings in the literature, using an *n*-back task in our study, we also did not observe a speed effect on cognitive performance, while motor parameters were however adapted to the different speeds. We also did not see very much task interference in most of the tested onset regimes. There was a tendency that reactions are a little delayed, when the stimulus was presented at the moment when one foot hit the ground (0% and 50% condition). In the 0% condition the stimulus is linked to the right foot and the right hand is used for the response, where as in the 50% condition stimulus link (left foot) and response (right hand) are contralateral. At the moment it remains unclear, whether this observed difference between the 0% and the 50% condition can be interpreted as an effect of motor response compatibility as reported by other researchers. Clear ipsilateral and contralateral limb coordination effects on reaction time have been described (e.g., Fujiyama, Garry, Martin, & Summers 2010, Miller & Gerstner 2013) as well as cross-talk effects in bimanual movements for flexion versus extension at critical reaction-time points (Kennedy, Wang, & Shea 2013). On the other hand, response onset could be linked with certain events in the rhythmic arm movement. Killeen and colleagues already showed that rhythmic arm swing while treadmill walking was affected by additional cognitive load, which could have corrupted RT measurements in a Stroop task (Killeen et al., 2017). In our study, subjects did not show the full amplitude of arm swing but rather suppressed arm movements due to holding the response box. Nevertheless, we cannot rule out that the lateralized cyclic activity of legs and arms might have affected the lateralized motor response. A follow-up experiment would be required to clarify to which extent these effects are relevant.

We observed the fastest RTs in the 75% and the Later condition. According to the different models presented in the introduction, we see our overall result profile as an indication that the processing requirements for walking are indeed different across the stride (*Temporal Fixed Binding to motor task*). However, the faster RTs under 75% condition are opposing to Lajoie et al. (1993), who observed slower RTs in one-leg stance compared to double-support phase. One major difference between both studies is that we had subjects walk on a treadmill in our experiment whereas Lajoie et al. used free overground walking. This difference might lead to different coordination/control requirements (Lee & Hidler 2008). Nonetheless, given that this observation only shows up as a trend in our data, we admit that further research is required to secure these phenomena. Even though many open questions remain, empirical evidence does not give any indication so far to dismiss the idea of a fixed cycle-linked alteration of processing requirements.

The main finding of an impaired cognitive processing is when stimuli appear earlier than expected. We are convinced that this empirical effect is robust, since it remained visible across a substantial range of walking speeds and thus, movement and stimulus frequencies. Different from many other comparable motor-cognitive dual-task studies (e.g. Kline et al. 2014, Nascimbeni et al. 2015, Penati et al. 2020, Wrightson et al. 2016), we counterbalanced the sequence of experimental conditions across subjects as far as possible in order to avoid systematic sequence effects. Nevertheless, these variations still affect the data as unsystematic error variance, diminishing effect sizes. Additionally, we also tried to reduce error variance by eliminating sequence effects, i.e. learning effects, by a non-linear regression. This allows us to integrate data across changing levels of expertise while excluding the variance due to the changing performance level itself.

Since the specific RT prolongation in the Earlier condition is, in principle, visible in each speed condition and statistically significant in the integrated analysis across all speeds despite the larger error variance, we consider it as a robust empirical effect which is worth looking for a conceptual interpretation.

The overall results are well in line with the aforementioned model of a *Triggered Interruption*. However, in conditions when the stimulus presentation is at a fixed location in the step cycle, a *scheduled temporal interleaving of processes* is possible impeding negative effects on performance. The idea of a preplanned allocation of processing resources is well in line with the concept of *time-based expectancies* as reported by Aufschnaiter, Kiesel, and Thomaschke (2018). They showed that subjects pre-allocate processing resources according to expectancies on the temporal course of events derived from previous practice experience.

According to our logic outlined in the introduction, we would need to interpret the observed absence of any performance decrement in the Later condition (violation of C2) as indication for a prescheduled resource allocation. However, why should the Later condition be different than the Earlier condition since both share the crucial property that the temporal location of the stimulus changes from stride to stride? We think that this seemingly contradictory result does not necessarily speak against the idea of a prescheduled resource allocation also in the Later condition. In this condition, the stimulus appears 5% of a stride cycle later than expected, amounting to a delay of approx. 100 ms in the 5 km h^–1^ condition. It may be possible that, in expectancy of the upcoming stimulus, the controller has scheduled a reduction of resources for the motor task also in this case. Importantly, this reservation still lingers on for a while. When delays are not too long, the stimulus may still come-in sufficiently timely to have the necessary share of processing resources in the reserved time window. However, this interpretation is still purely speculative at the moment. Again, future experiments that systematically change the delay of the late-coming stimulus might be useful to test how sustainable this interpretation is.

## Conclusion

We have developed different ideas on how resources might be allocated to both tasks when a cyclic and continuous motor task is performed in parallel with an intermittent cognitive task. We have manipulated the onset regime of stimuli for the cognitive task. We found support for the idea of a preplanned allocation of processing resources, which is well in line with the concept of *time-based expectancies* as introduced in the context of task-switching. Our study gives indication that this may also play a role in the control of cyclic cognitive-motor dual tasks. So far, our interpretation heavily relies on the observation that we see interference when temporal expectancies are useable compared to when they are potentially violated. We will need further studies to secure this effect.

## Data Accessibility Statement

Dataset is available on Zenodo (https://zenodo.org/badge/DOI/10.5281/zenodo.4028441.svg).
